# Comparative Anticancer Potential of Green Tea Extract and Epigallocatechin-3-gallate on Breast Cancer Spheroids

**DOI:** 10.3390/foods13010064

**Published:** 2023-12-23

**Authors:** Ronimara A. Santos, Heloisa Rodrigues Pessoa, Julio Beltrame Daleprane, Giselle Pinto de Faria Lopes, Danielly C. Ferraz da Costa

**Affiliations:** 1 Laboratory of Physiopathology and Biochemistry of Nutrition, Nutrition Institute, Rio de Janeiro State University, Rio de Janeiro 20550-013, Brazil; ronimaras@gmail.com (R.A.S.); mrs.pessoa@gmail.com (H.R.P.); 2 Laboratory for Studies of Interactions between Nutrition and Genetics, Nutrition Institute, Rio de Janeiro State University, Rio de Janeiro 20550-013, Brazil; juliobd@gmail.com; 3 Almirante Paulo Moreira Institute of Sea Studies, Division of Natural Products, Department of Marine Biotechnology, Arraial do Cabo 28930-000, Brazil; giselle.faria@gmail.com

**Keywords:** phytochemicals, green tea, food matrix, breast cancer, 3D culture

## Abstract

Despite advances in diagnosis and therapy, breast cancer remains the leading cause of death in many countries. Green tea (GT) has been proposed to play a crucial role in cancer chemoprevention. Although extensive research has been conducted on GT phytochemicals, most experimental studies concentrate mainly on commercial formulations or isolated catechins. This study presents a comparative investigation into the anticancer properties of green tea extract (GTE) and epigallocatechin-3-gallate (EGCG) in a three-dimensional (3D) MCF-7 breast cancer cell culture. MCF-7 spheroids were exposed to GTE or EGCG, and effects on 3D culture formation, growth, cell viability, and migration were examined. GTE inhibits cell migration and the formation of breast cancer spheroids more effectively than EGCG, while inducing more pronounced morphological changes in the spheroids’ structure. These findings suggest that the food matrix improves GTE effects on breast cancer spheroids, supporting the hypothesis that a mixture of phytochemicals might enhance its anticancer potential.

## 1. Introduction

Cancer has emerged as a prevalent chronic non-communicable disease in modern society. According to the International Agency for Research on Cancer, with an estimated 2.3 million new cases (11.7%) in 2020, female breast cancer has surpassed lung cancer as the most frequently diagnosed malignancy. Despite advances in diagnosis and therapy, breast cancer still ranks first in mortality across 110 countries worldwide [1]. From the perspective of a global-scale public health challenge, the goal of basic oncology research is to better understand the molecular processes involved in carcinogenesis so that novel chemopreventive and chemotherapeutic strategies can be developed [2,3].

The anticancer potential of naturally occurring compounds on plant-based foods offer opportunities in the search for therapeutic approaches for cancer management and prevention. In this scenario, the chemopreventive potential of green tea (GT) (Camelia sinensis) appears to be a promising target in breast cancer research [4]. The processing steps by which GT is obtained comprise the fixation, rolling, and drying of fresh leaves [5]. The main composition of GT includes phytochemicals such as caffeine, theobromine, catechins, kaempferol, theaflavins, L-theanine, quercetin, and myricetin. All these variations highlight the GT food matrix’s diversity and complexity [6,7].

The identification of the main biological targets of GT will support its future application as a therapeutic agent [8]. Biological activities of GT on breast cancer have been shown in in vitro and in vivo studies, and they appear to be involved in the modulation of crucial processes in tumor growth, maintenance, and progression [9]. However, it should be noted that many studies use standardized extracts or isolated phytochemicals. Interactions among food components can alter their properties, causing them to behave differently than in their isolated forms, and this phenomenon is commonly referred to as the “food matrix effect” [10,11].

The term food matrix refers to a complex set of nutrients and non-nutrients that interact physical and chemically to influence the release, digestibility, stability, and absorption of many food compounds, thereby altering their bioavailability and bioaccessibility [12,13]. It has previously been reported that caffeine, a natural component of GT, increases epigallocatechin-3-gallate (EGCG) bioavailability [14]. These findings reflect the growing interest in investigating the anticancer effects of dietary matrix components to determine whether an isolated molecule could replace matrix-rich phytochemicals in providing health benefits [15,16].

The two-dimensional (2D) cell culture model has been supporting basic cancer research for over a century. However, because tissues and organs are three-dimensional, the limitations of employing 2D cultures have begun to be questioned [17]. The 2D cultured cell lines lose their polarity, leading to changes in the response of biological processes. Other disadvantages include unlimited access to oxygen, nutrients, metabolites, and cellular signaling molecules, as well as alterations in gene expression [18,19]. It is crucial to consider that these interactions play an essential role in biological events related to carcinogenesis, including differentiation, proliferation, and responsiveness to therapeutically relevant compounds [20].

Despite the understanding regarding the synergy of bioactive compounds, experimental studies evaluating the effects of whole natural foods versus isolated phytochemicals on cancer cells are still limited. The present investigation elucidates the comparative anticancer potential of green tea extract (GTE), obtained from Camellia sinensis infusion, and its major catechin, epigallocatechin-3-gallate (EGCG), on 3D MCF-7 culture. Our findings indicate that GTE inhibits cell migration and the formation of breast cancer spheroids more effectively than EGCG, while also inducing more pronounced morphological changes in the spheroids’ structure.

## 2. Materials and Methods

### 2.1. Chemicals and Reagents

All reagents were of analytical grade, and the water was obtained from a Milli-Q Millipore system (Bedford, MA, USA). From Thermo-Fisher Scientific (Saint Louis, MO, USA), we obtained trypsin, antibiotics (penicillin and streptomycin), Alamar Blue^®^, fetal bovine serum (FBS), Dulbecco’s Modified Eagle’s Medium (DMEM), and Mammary Epithelial Growth Supplement (MEGS). The catechin standards were purchased from Indofine Chemical Co. (Hillsborough, NJ, USA). Agarose was purchased from KASVI (São José dos Pinhais, Brazil). Mitomycin C and EGCG were purchased from Sigma-Aldrich Chemical Co. (Saint Louis, MO, USA).

### 2.2. Green Tea Extract

A commercial brand of oven-roasted green tea (*Camellia sinensis*) was acquired in a local market in Rio de Janeiro, Brazil. An aqueous infusion was prepared from dried leaves in the proportion of 1 g:40 mL at 80 ± 2 °C for 5 min. The temperature was measured with a thermometer positioned in the middle of the container, and the mixture was kept under constant agitation to increase the extraction efficiency of phenolic compounds [21]. The extract was cooled to room temperature, filtered through filter paper, centrifuged, and then lyophilized (Terroni LS 3000, São Paulo, Brazil). The dry extract was stored at −20 °C and protected from light. GTE was diluted in culture medium and filtered via a 0.22 µm filter to ensure sterility.

### 2.3. Characterization of Green Tea Extract Polyphenols by HPLC

The catechin content in GTE was determined by High-Performance Liquid Chromatography (HPLC) analysis. The HPLC system (Shimadzu^®^, Kyoto, Japan) included two LC-20AD pumps, automatic injector SIL-20AHT, diode array detector SPD-M20A, system controller CBM-20A, and degasser DGU-20A5. Chromatographic separation of catechins was achieved using a reverse-phase column C18 (5 µm, 250 mm × 4.6 mm, Kromasil^®^, Darmstadt, Germany). The mobile phase consisted of a gradient of 0.3% formic acid and 1% acetonitrile in water (eluent A) and 1% acetonitrile in methanol (eluent B), at a flow rate of 1.0 mL/min. Prior to injection, the column was equilibrated with 18.2% B. After sample injection, the solvent composition changed to 20.2% B in 1 min, 43.4% B in 18 min, and 85.9% B in 23 min, and remained constant until 30 min between injections, and 10 min intervals were used to re-equilibrate the column with 18.2% B. The eluent was monitored by DAD at 210 and 280 nm. The injection volume was 10 µL. The identification of catechin (C), epicatechin (EC), epigallocatechin (EGC), epigallocatechin-3-gallate (EGCG) and epicatechin gallate (ECG) was performed by a comparison with the retention time and absorption spectrum of the respective standard. Quantification was performed by external calibration. Data were acquired using LabSolutions software (Shimadzu Corporation^®^, Sydney, Australia, version 5.82, 2015).

### 2.4. The 2D and 3D Cell Culture

The human breast epithelial carcinoma cell lines MCF-7 and MCF-10A were obtained from the Rio de Janeiro Cell Bank (RJCB, RJ, Brazil). The tumoral cells MCF-7 were cultured in DMEM containing 4.5 g/L glucose supplemented with 10% fetal bovine serum and 1% of penicillin/streptomycin and maintained at 37 °C in a humidified atmosphere containing 5% CO_2_. The non-tumoral cells MCF-10A were cultured under identical conditions, with the addition of Mammary Epithelial Growth Supplement (MEGS).

To develop 3D structures, a previously reported protocol was adopted with modifications [22]. A spheroid initiation procedure was carried out using a single-cell suspension by enzymatic dissociation with trypsin 0.05%. To avoid cell attachment, 5.0 × 10^3^ cells/mL or 1.0 × 10^5^ cells/mL, respectively, for MCF-7 and MCF-10A, were seeded in 1% agarose-coated 96-well plates containing DMEM, as described above. Plates were centrifuged at room temperature for 10 min at 400× *g* to allow cell sedimentation before being incubated for 96 h in a humidified atmosphere with 5% CO_2_ at 37 °C. Spheroids with average diameters of 300 to 450 µm were used for the experimental treatments.

### 2.5. Spheroid Growth Pattern Follow Up

The spheroids of MCF-7, obtained as described, were monitored for fifteen days. Half of the culture medium was replaced every 3 or 4 days. The images were captured every 24 h with 10× magnification, using Lumenera^®^ software version 4.0. Image areas were quantified using ImageJ^®^ software version 1.43p.

### 2.6. Treatment of Spheroids with GTE or EGCG

MCF-7 spheroids were exposed to GTE, by replacing half of the final media volume in each well. Spheroids were exposed to GTE concentrations of 162 µg/mL, 324 µg/mL, 648 µg/mL, 1296 µg/mL, and 2592 µg/mL for 24 h and 48 h. For EGCG treatment, the studied concentrations (62.5 µg/mL, 31.2 µg/mL, 16.6 µg/mL, and 7.8 µg/mL) were diluted in the culture medium in each well from a stock solution of 10 mg/mL.

### 2.7. Cell Viability Assay

For access cell viability, the Alamar Blue^®^ (Invitrogen, Carlsbad, CA, USA) assay protocol was adopted [23]. Following treatment with GTE or EGCG, spheroids were transferred to a new non-coated plate, which was then centrifuged (400× *g*, 10 min, room temperature). The medium was subsequently discarded, and the spheroids underwent two washes with phosphate-buffered saline (PBS). Afterward, the spheroids were stained with 10% of Alamar Blue^®^ reagent in DMEM (2% SFB) for 24 h at 37 °C within a humidified atmosphere containing 5% CO_2_. Following this incubation period, 200 µL of the supernatant was transferred to a new plate and the absorbance was read by spectrophotometry Biochrom^®^ Asys UVM340 (570–600 nm). The resulting data were expressed as a percentage of cell viability considering the control condition as 100%.

### 2.8. Migration Assay

For the migration assay, a reported protocol was adopted with adaptations [24]. After 4 days of culture, spheroids (300 to 450 µm diameter) were individually transferred to a 24-well plate, with each well containing varying concentrations of GTE (162 µg/mL, 324 µg/mL, 648 µg/mL) or EGCG (31.2 µg/mL, 16.6 µg/mL and 7.8 µg/mL). To control cell proliferation, FBS was reduced to 2% and a proliferation inhibitor, mitomycin-C (0.5 µg/mL), was added. Images were captured at 0 h, 24 h, 48 h, and 72 h using a 10× objective using Lumenera^®^ software.

### 2.9. Statistical Analyses

Data were expressed as mean ± standard deviation (SD) from at least three independent experiments. We confirmed the data for the normal distribution and homoscedasticity of the variance using a Shapiro–Wilk test, and then the groups were compared using one-way analysis of variance (ANOVA). In all cases, a *p* value < 0.05 was accepted as statistically significant using GraphPad Prism Software v6.01 (San Diego, CA, USA).

## 3. Results

### 3.1. GTE Affects the Morphology and Growth Pattern of Breast Cancer Spheroids

To characterize the growth kinetics and cell density required, beginning with a spheroid with 300–400 µm diameter, preliminary experiments were carried out. It was observed that spheroids demonstrated a dark core compared to the cortical area between the 6th and 7th days in culture when using optical microscopy. The dark core increased until the 10th day in culture, when it stabilized, without affecting the integrity of the cortical area. The presence of these structures observed between days 10 and 14 made the spheroids assume a less spherical arrangement, with heterogeneous edges. The spheroids showed a linear growth pattern for the first seven days of culture, before reaching a plateau on the 8th day (Figure S1).

Aiming to assess the suitability for application in cell culture, we further determined the content of the four primary catechins found in GT leaves (Table S1). The chromatographic analysis revealed the presence of four major catechins in GTE, with epigallocatechin-3-gallate (EGCG) and epigallocatechin (EGC) being the most abundant, accounting for 41% each. This was followed by epicatechin gallate (ECG) at 11% and Catechin (C) at 7%.

**Figure 1:**
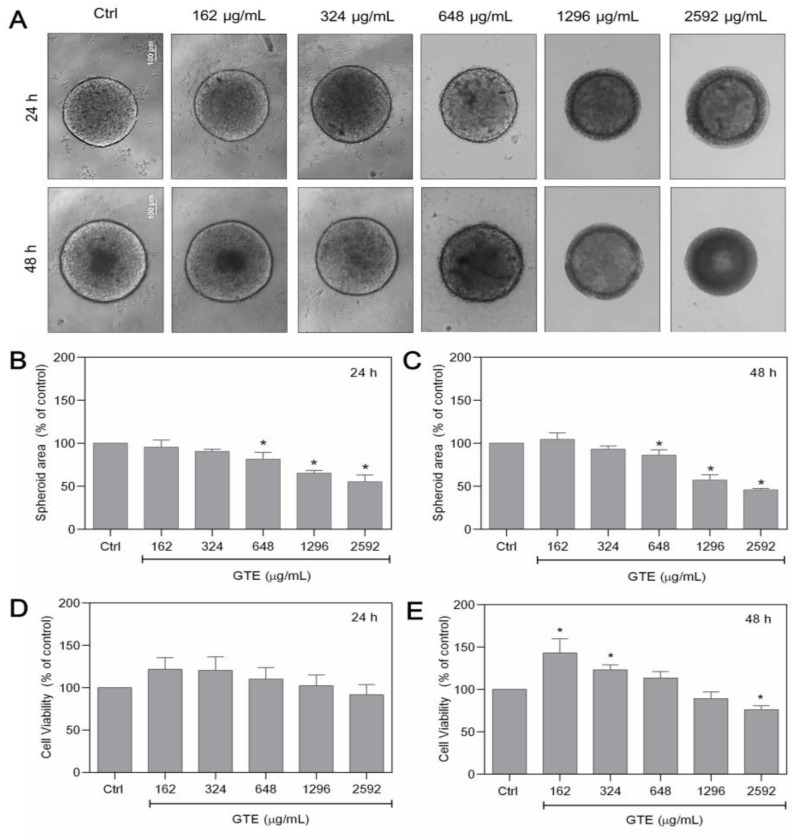
GTE changes the morphology and the size of MCF-7 spheroids. Spheroids were exposed to different GTE concentrations for 24–48 h (**A**). Spheroid’s area was measured (**B**,**C**). Cell viability was assessed using Alamar Blue^®^ (**D**,**E**). Scale bars: 100 µm. Experiments were performed in triplicate and results were expressed as % of controls; * indicates significant difference from controls (*p* < 0.05).

To assess the anticancer potential of GTE, spheroids were cultured with increasing GTE concentrations (162–2592 µg/mL) for 24–48 h and the impact on morphology, cell viability, and spheroid area were examined (Figure 1).

Exposure to GTE resulted in a significant modification of the spheroid’s basic structure. After 24 h, morphological differences in the cortical proliferating area were observed, which appeared less compact and acquired a granular appearance. In the last two highest GTE concentrations, the spheroids presented a border at the core, decreasing its area in a concentration-dependent manner. However, after 48 h, the dark core area was observed in the control and in the first concentration treatment with GTE, and was absent up to 324 µg/mL. The core border increased at the highest GTE concentration, as observed at 24 h (Figure 1A). Starting from 648 µg/mL of GTE, spheroids showed a significant total area reduction in a concentration-dependent manner, reaching 55% ± 8 and 46% ± 2 during 24 h and 48 h, respectively (Figure 1B,C). Modifications in the compactness of the spheres were also observed after 24 h and 48 h of treatment. Spheroids became friable at concentrations greater than 648 µg/mL of GTE. At this point, we observed that some spheroids could crumble with mechanical manipulation. This phenomenon became more common as the concentration of GTE increased. Exposure to 2592 µg/mL had a significant effect on the spheroid compactness, and the structures were entirely disrupted by simple manipulation.

There was no significant reduction in cell viability in MCF-7 spheroids treated with GTE for 24 h. Nevertheless, after 48 h, the cell viability of spheroids exposed to GTE at 162 µg/mL (143% ± 16.7) and 324 µg/mL (123% ± 7.5) increased significantly compared to the control. However, a significant decrease in cell viability was observed when spheroids were cultivated with the highest GTE concentration (76% ± 4.6) (Figure 1D,E).

### 3.2. GTE Had No Cytotoxic Effects on MCF-10A Cells

**Figure 2:**
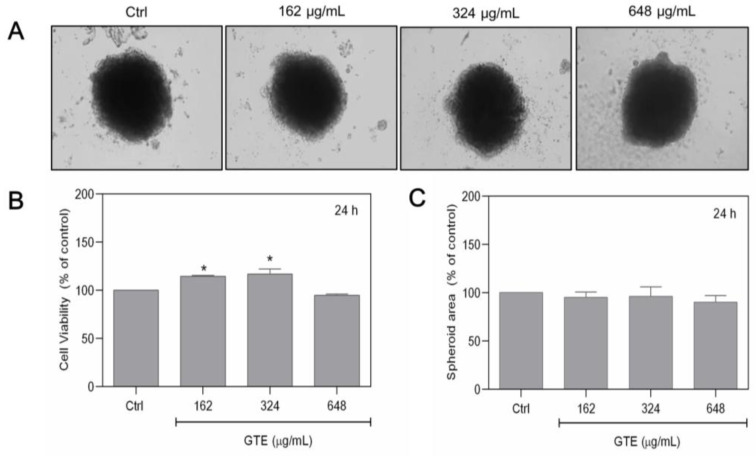
Non-tumoral MCF-10A spheroids were not affected by GTE. Here, 1.0 × 10^5^ cells/mL were seeded by centrifugation in agarose-coated plates in the presence of different concentrations of GTE (**A**). Cell viability was assessed by Alamar Blue^®^ (**B**). The areas of spheroids were quantified (**C**). Experiments were performed in triplicate on two independent experiments. * indicates significant difference from control (*p* < 0.05).

To determine whether the effects of GTE are selective to tumoral cells, experiments were also carried out on non-tumoral MCF-10A breast spheroids. MCF-10A cells had a comparable ability to spontaneously form spheroids, similar to MCF-7 cells. However, since it is a non-tumoral cell line, its growth is slower, and so the previous cell density applied to MCF-10A was insufficient to form spheroid structures. As a result, 1.0 × 10^5^ cells/mL was chosen as the initial seeding density. Figure 2A exhibits the overall appearance of the 3D. MCF-10A spheroids were exposed to a range of GTE concentrations (162–648 µg/mL), and the cell viability and spheroid area were assessed. Our findings indicate that GTE had no cytotoxicity in non-tumoral spheroids (Figure 2).

### 3.3. ECGC Treatment Does Not Promote the Same Effects as GTE

**Figure 3:**
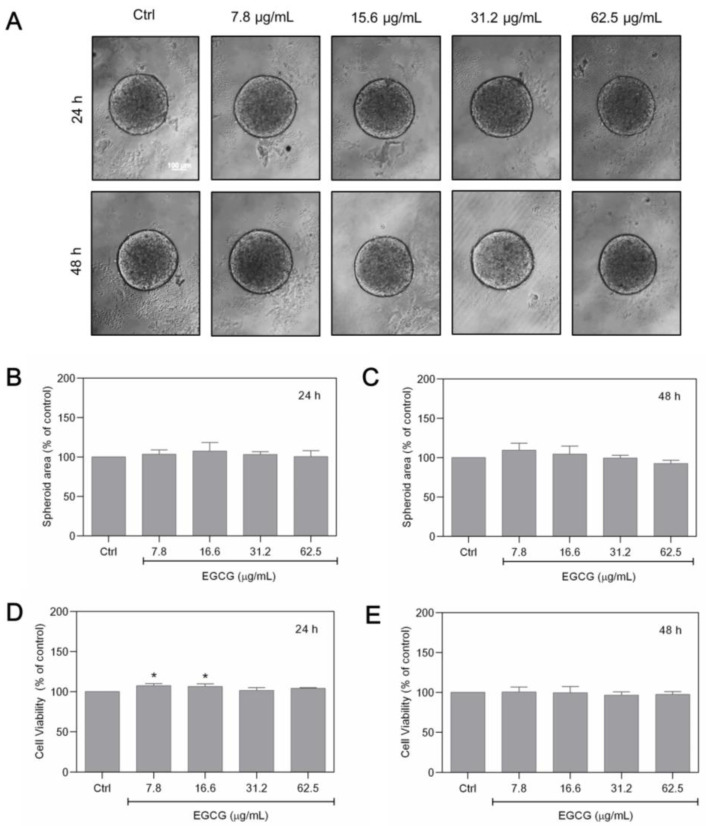
EGCG does not affect MFC-7 spheroids. Spheroids were exposed to different EGCG concentrations for 24–48 h (**A**). The area of the spheroids was measured (**B**,**C**). Cell viability was assessed by Alamar Blue^®^ (**D**,**E**). Scale bars: 100 µm. Experiments were performed in triplicate and results were expressed as % of controls; * indicates significant difference from controls (*p* < 0.05).

After determining the effects of GTE on the area and structure of MCF-7 spheroids, we investigated whether these effects could be achieved with isolated catechins, and therefore the same experiments were performed using epigallocatechin-3-gallate. EGCG was tested in seriated concentrations ranging from 7.8 to 62.5 µg/mL, which corresponded to the range of EGCG contained in the GTE concentrations used. Figure 3 shows the effects of EGCG on morphology, cell viability, and area in spheroids cultivated for 24 and 48 h.

When compared to GTE, EGCG appears to have no influence on the morphology of these cells, which all had a circular cortical border (Figure 3A). In addition, no statistically significant differences were observed in the diameter of the treated spheroids compared to the control (Figure 3A–C). The Alamar Blue^®^ viability assays also did not show a significant decrease in cell viability at the EGCG concentrations tested (Figure 3D,E).

### 3.4. GTE Inhibits Spheroids’ Formation

**Figure 4:**
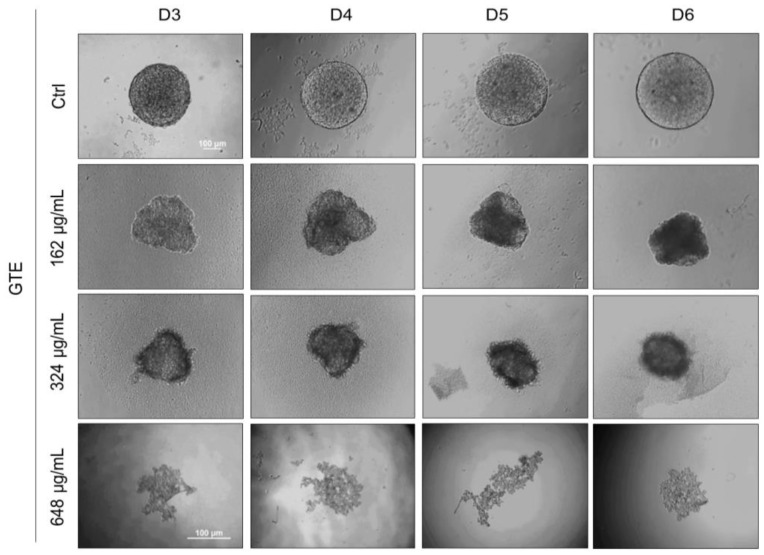
GTE impairs MCF-7 spheroid formation. Here, 5.0 × 10^3^ cells/mL were seeded by centrifugation in agarose-coated plates in the presence of different concentrations of GTE, as indicated. Cells were monitored for 6 days and images were captured every 24 h. Scale bars: 100 µm. Assays were performed in triplicate, in three independent experiments.

Based on the previous results, we hypothesize that GTE could interfere with the formation of spheroids and tumorigenesis. A novel experiment was conducted whereby spheroids were generated in the presence of GTE. As shown in Figure 4, prior exposure to GTE in the early stages of 3D cell culture resulted in abnormal spheroids, less compact aggregates, irregular edges, and smaller sizes compared to the control. The formation of spheroids was impaired at 648 µg/mL of GTE and cellular aggregates were observed rather than the robust structures seen previously. The formation of spheroids in the presence of lower concentrations of EGCG (up to 15.2 µg/mL) was apparently not affected.

### 3.5. GTE, but Not EGCG, Reduces Spheroids’ Cell Migration

**Figure 5:**
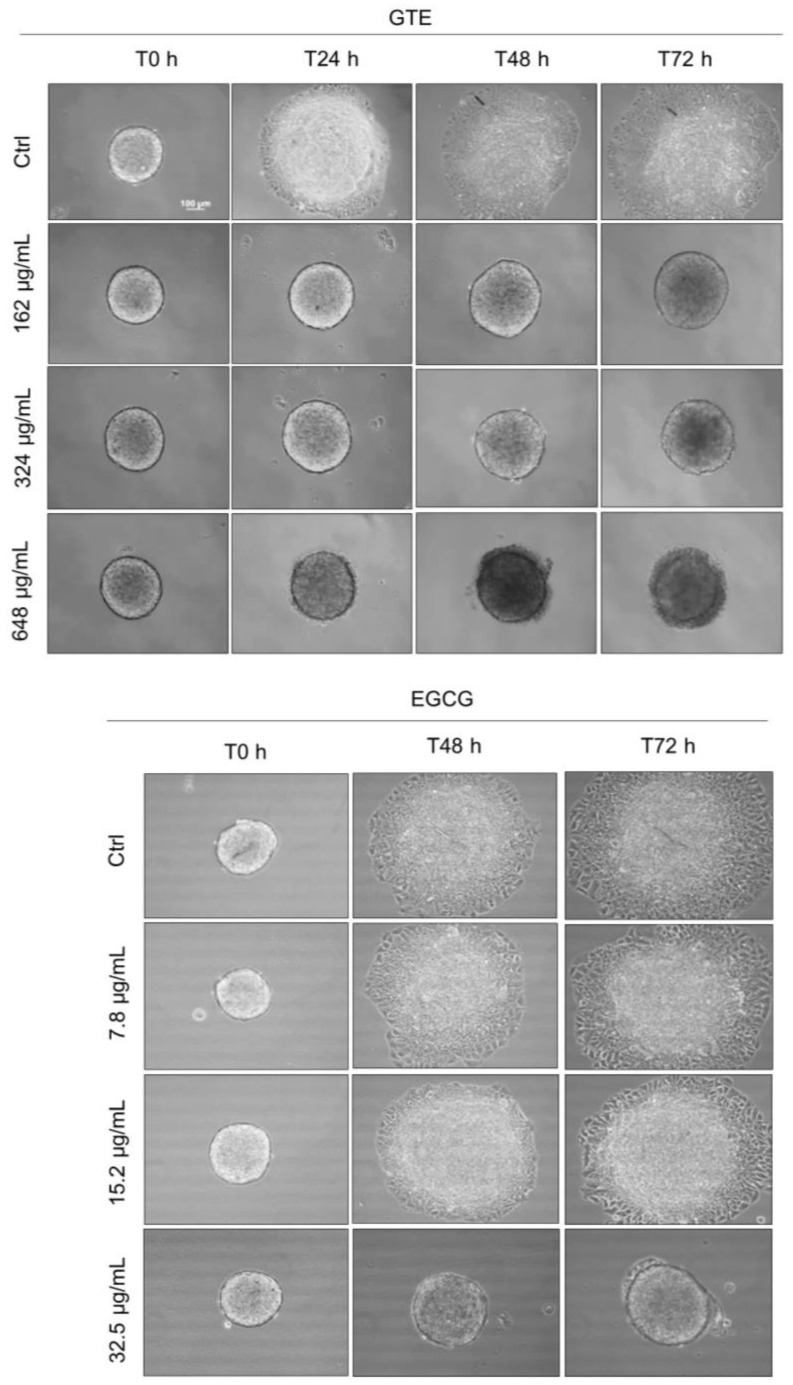
GTE inhibits MCF-7 cells’ migration from spheroids. Spheroids with four-day culture were individually transferred to a non-agarose-coated plate and exposed to different concentrations of GTE or EGCG, in the presence of 2% SFB and 0.5 µg/mL mitomycin C. Images were captured at 0 h, 24 h, 48 h, and 72 h. Scale bars: 100 µm. Assays were performed in triplicate, on three independent experiments.

Given the potential role of migratory capability in tumor progression, we investigated the ability of GTE in suppressing the migration of MCF-7 cancer spheroid cells. On the fourth day, the spheroids were transferred individually to a 24-well plate (not covered with agarose) and exposed to different concentrations of GTE or EGCG. To control cell proliferation, SFB was reduced to 2% and a cell proliferation inhibitor (mitomycin C at 0.5 mg/mL) was added. GTE inhibited cell migration at all test conditions (Figure 5). Even though 162 μg/mL of GTE had no effect on spheroid viability, structure, or area, it was enough to fully prevent cell migration. As in the control group, the spheroids lost their capacity to attach to the plate surface. There was no effect observed in ECGC-treated groups with concentrations less than 15.2 µg/mL.

## 4. Discussion

Plant-based functional foods are a valuable source of anticancer bioactive compounds and have gathered considerable interest in the field of cancer research [25]. The hypothesis that phytochemicals may mitigate the risk of cancer is based on evidence from epidemiological observational studies, which demonstrate a correlation between the consumption of plant-derived foods and a reduced susceptibility to numerous chronic diseases, including cancer [26].

Green tea (GT), a widely consumed beverage worldwide, has demonstrated great promise as a chemopreventive agent by efficiently inhibiting mutagenesis and tumor promotion and progression [27,28]. Previous research showed that GT intake has been associated with the prevention of tumor development, including lung, colon, esophageal, oral, stomach, small intestine, kidney, pancreas, and mammary gland cancers [29]. Tea catechins have demonstrated significant anticancer effects either in vitro or in vivo [30,31,32,33]. However, evidence suggests that exposure to compounds as they occur naturally in food has a significant influence on antioxidant potency, bioavailability, and safety [25]. The complexity of the food matrix impacts the release, digestibility, and stability of several dietary components, including phenolic compounds [34]. As a result, experimental investigations that use the dietary matrix instead of isolated compounds are required to promote new perspectives on natural products’ benefits for chemoprevention. To investigate the impact of the food matrix on the effects of GT on breast cancer, our study focused on examining the effects of green tea extract (GTE) or its primary catechin, EGCG, on MCF-7 3D culture.

Over the last century, two-dimensional (2D) model cultures have supported experimental cancer research [35]. Tissues and organs, on the other hand, are three-dimensional, and the advantages of two-dimensional cultures are being questioned. It has been established that 2D cell cultures have limited access to oxygen, nutrients, metabolites, cellular signaling molecules, and changes in gene expression, and hence do not accurately replicate the natural structure of tumors, particularly cell–cell interactions [18]. It should be noted that these interactions are required to respond to chemicals with therapeutic aims, emphasizing the significance of research on tridimensional culture models [36].

For this study, MCF-7 cells were chosen because of their spontaneous ability to generate spheroids due to the presence of strong cell–cell interactions. This ability to grow independently of anchorage is even considered a classic predictor of tumorigenicity [37]. The morphology of cells grown using the 3D technique differs significantly from that of cells grown using the standard monolayer method. MCF-7 cells develop in clusters in 2D culture and spread across the surface until completely confluent. MCF-7 cells aggregate into a spherical shape that is tightly packed into the 3D arrangement. We initially examined the growth pattern of MCF-7 spheroids and observed changes in their spherical morphology over time. A similar phenomenon was noted when MCF-7 cells were cultured in 3D on hydrophobic plates [38]. Our findings are consistent with the formation of breast cell colonies in 3D culture, specifically within the mass class group, characterized by colonies exhibiting disorganized nuclei and strong cell–cell interactions [39].

MCF-7 spheroids were exposed to different concentrations of GTE or EGCG. GTE production considered the influence of time and temperature on polyphenol quality and replicated previously established optimal conditions [40]. The procedure also aimed to replicate the standard tea preparation process used by customers, employing the binomial 80 °C/5 min [21]. The results presented in this study showed that GTE, but not ECGC, selectively promotes changes in the size and morphology of breast cancer spheroids, leading to a weakening of their three-dimensional structure. The reduction of the necrotic nucleus observed in 48 h at 648 µg/mL of GTE suggests a reduction in proliferation status and aggressiveness, corroborated by a significant decrease in cell viability in 48 h. Similar results were found in the literature about the effects of a commercial GTE (EFLA942) on the growth, viability, and metabolism of WiDr colon cancer cells. At the highest concentrations (100 µg/mL), GTE also disrupted the spheroidal structure, which could be separated by simple manipulation. Additionally, changes in the adhesion behavior of treated cells consistent with a reduction in the formation of colonies were also reported [41]. GTE’s ability to reduce spheroid growth has been demonstrated in 3D cultures of 4T1 murine mammary carcinoma cells [42] and prostate LNCaP [43]. We also demonstrated that spheroid formation was impaired in a GTE-containing medium, leading to irregular aggregates. Therefore, it would be helpful to explore the impact of GTE on adhesion molecules that facilitate cell-substratum attachment, such as integrins [44,45].

Cancer prognosis is negatively impacted by the spreading of malignant cells into the bloodstream, resulting in the formation of metastatic lesions [46]. This mechanism might be inhibited or controlled to delay the progression of the disease [39]. Hence, we investigated the effects of GTE on cell migration from MCF-7 spheroids. GTE suppresses MCF-7 spheroid migration, which was not as evident in EGCG-treated spheroid cultures. Previous studies have shown evidence that both the phenolic and non-phenolic components of GT had the ability to prevent the migration of cancer cells by triggering the disintegration of microtubules [47]. GT might have a modulatory action on protein kinases. In the presence of 50 µM of each of the catechins with the gallate group (CG, ECG, EGCG), the formation of A172 spheroids in agar was inhibited, and this effect was attributed to the suppression of the activity of PDGF-R tyrosine kinase, a receptor involved in signaling processes of angiogenesis, proliferation, and cellular migration [37]. The treatment with EGCG (10–50 µg/mL) resulted in a dose-dependent decrease of up to 66.5% in the migration of 2D SW480 cells, and this effect was associated with a significant reduction in vimentin and an increase in E-cadherin [48]. Indeed, pre-incubation with GTE (80 μg/mL) and EGCG (60 μmol/L) significantly reversed the impacts of TGF-β on TGF-β-induced EMT in 2D Hela and SiHa cells, reducing vimentin expression and increasing E-cadherin expression [49]. The GTE regulation of E-cadherin expression may explain, at least in part, the phenomena of migratory inhibition found in the MCF-7 spheroids in our study. Given the variations in compound responses in 2D/3D cultures, it may be relevant to study whether the effect of GTE on these adhesion molecules and mesenchymal epithelial transition markers is reproducible in spheroids.

Another hypothesis suggests the involvement of GTE in the expression of matrix metalloproteinases (MMPs). MMPs are implicated in the degradation of intratumoral basement membranes, which loosens cell–cell connections and allows cells to migrate into the bloodstream or lymphatic system [50]. For instance, in patients with breast cancer, elevated plasma concentration and activity of MMP-2 and -9 have been identified and these biomarkers are associated with an increased risk of disease progression [51]. The transition of a primary tumor into a metastatic stage can also be regulated through the generation of reactive oxygen species (ROS), which are abundantly produced in rapidly proliferating tumor cells. The effect of GTE (20 µg/mL) and EGCGC (7.8–125 µg/mL) in reducing MMP-3, -8, and -9 was also observed in a three-dimensional co-culture of fibroblasts and U937 monocytes, with the authors highlighting the superior effect of GTE over EGCG [52]. The GT mechanisms of action on MMPs can also be seen through the control of their translation, and in this field the modulation of microRNAs may offer a therapeutic alternative. The exposure of LNCaP prostate cancer spheroids to 100–1000 µg/mL of GTE for 48 h led to an increase in miR-181a expression [43]. MiR-181a are known to inhibit migration and angiogenesis through the regulation of MMP-14 in SK-3 breast cancer cells [46]. Also, a positive correlation between ROS production and increased MMPs expression has been reported [53]. The regulation of ROS production by the phenolic compounds of GT is a well-known capability [2].

Moreover, from a toxicological standpoint, it is generally safer to obtain bioactive compounds from plant-based foods than from concentrated nutritional supplements. For example, GT appears to be well tolerated, with minimal side effects reported (0.6%). Cases of toxicity are closely related to the total concentration of catechins provided, and in this context the use of a matrix increases safety [54]. Preclinical studies have shown that animals exposed to GTE with a lower concentration of catechins (<40% *w*/*w*) experience fewer adverse effects (weight loss, irritation of the gastrointestinal mucosa, ulceration, necrosis) than those exposed to isolated catechins or extracts with high doses of these substances. Human studies suggest that GT is better tolerated in beverages than in capsules or bolus, since the latter increases the amount of free catechins in the bloodstream and the liver [55]. Under these considerations, our data revealed that the effects of GTE on MCF-7 spheroids were more pronounced than those of EGCG, highlighting the significance of using GT as its food matrix.

On the other hand, experimental research in oncology has been predominantly conducted using 2D culture systems during the last century, and it is important to emphasize that tissues and organs exist in a three-dimensional configuration [56,57]. It is recognized that 2D cultures do not accurately replicate the natural structure of tumors, particularly cell–cell interactions [18]. It should be stated that these interactions are essential to respond to compounds with therapeutic aims. In comparison to a prior investigation of cell viability in 2D culture, our results demonstrated that spheroids were less sensitive to GTE. Despite a cytotoxic effect on 2D culture, GTE treatment showed no effect on MCF-7 3D culture viability at the same concentrations [40]. Traditionally, 3D cultures have been shown to be more resistant to compound activity than monolayer cultures, which may explain the results found. Indeed, the packing density acts as a barrier to doxorubicin penetration in a multi-cellular layer colon cancer culture, reducing the drug’s activity [58]. In this context, 3D architecture would be a critical component in GTE penetration. This effect is associated, in part, with cell–cell and cell–extracellular-matrix interactions in the spheroid, which make drug penetration more difficult, and which is a process that is not observed in the 2D model [20]. In addition, the inherent differences in gene expression in 3D/2D culture also help in understanding this phenomena. Breast cancer spheroids (BT474, HCC1954, EFM192A) were more resistant to the drugs neratinib and docetaxel, due to the increase in both CYP3A4 activity and the expression of two membrane transporters associated with resistance (PGP and BCRP). It is widely recognized that the responses to compounds in the 3D model are less sensitive, as appears in the in vivo tumor [56]. Overall, cells naturally grow in a 3D environment, and this difference in sensitivity between 2D and 3D cultures suggested that spheroids would be useful for the pre-in vivo screening of natural compounds for cancer therapeutics [59]. These findings highlight the relevance of employing a 3D model in the research of bioactive compounds for therapeutic purposes.

Furthermore, it is important to consider the natural properties of therapeutic compounds. The comprehensive investigation of GT’s biological potential within the context of a complete food matrix is still limited. By focusing on mechanisms of action, the scientific hypotheses assume that biological activities of food matrices are due to the combination of phytochemicals, rather than an isolated agent. The mixture of a range of compounds present in whole foods could have additive or synergistic anticancer effects. Evidence suggests that exposure to compounds as they naturally occur in food has a major impact on their antioxidant power, bioavailability, and safety [16]. The complexity of the matrix components influences the release, digestibility, and stability of many food compounds, including phenolic compounds [34]. For this reason, experimental studies that apply the food matrix as an alternative to isolated compounds are necessary to support new perspectives on natural products’ benefits for chemoprevention. Multiple biological mechanisms have been proposed for isolated GT phytochemicals [60]. However, the potential of this plant-based food matrix needs to be further explored.

## 5. Conclusions

The incorporation of dietary phytochemicals represents a feasible, cost-effective, and easily applicable approach for chemoprevention. In this field, experimental research can provide valuable information regarding plant-based functional food properties. Our results suggest that GTE, derived from the food matrix, has a greater ability to inhibit cell migration and breast cancer spheroid formation, while also inducing more pronounced modifications in the morphology of 3D MCF-7 structures, in comparison to its primary catechin, EGCG. This study provides new insights into the significance of the food matrix, regarding the anticancer potential of GTE. Future research is required to explore plant-based food potential given the lack of comparative studies assessing whole natural foods versus isolated phytochemicals.

## Data Availability

Data is contained within the article or supplementary material.

## Supplementary Materials

The following supporting information can be downloaded at: https://www.mdpi.com/article/10.3390/foods13010064/s1, Figure S1: MCF-7 spheroids’ growth pattern; Table S1: Chemical composition of the GTE.
