# Supplementary material for: Comparative Anticancer Potential of Green Tea Extract and Epigallocatechin-3-gallate on Breast Cancer Spheroids
Source: Foods. 2023 Dec 23;13(1):64. doi: 10.3390/foods13010064 (PMC10778335; doi:10.3390/foods13010064)
Supplement: Supplementary file 1 [file foods-13-00064-s001.zip › foods-2746708-supplementary.pdf]

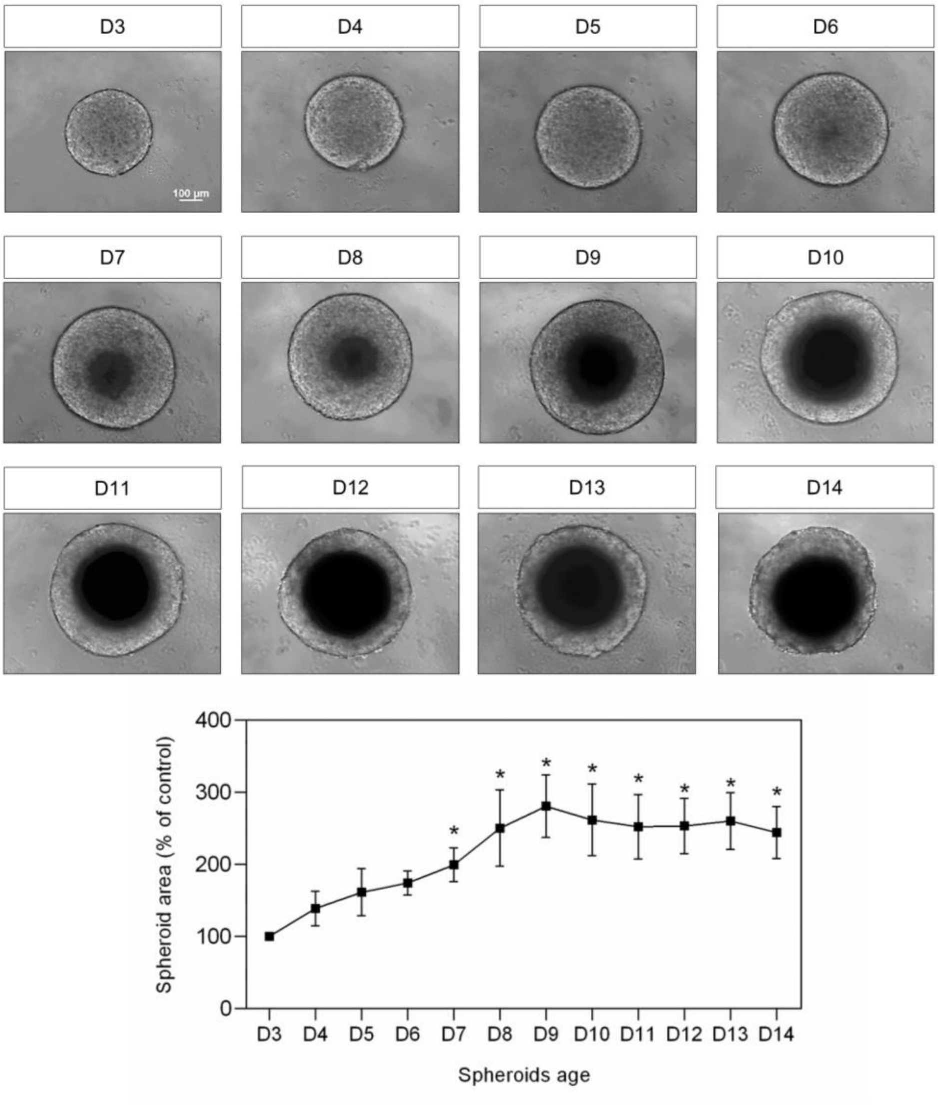

Figure S1. MCF-7 spheroids' growth pattern: 5.0×10^3 cells/mL were seeded by centrifugation in agarose-coated plates. The spheroids were followed up for 2 weeks, and the images were captured every 24 h. Image areas were quantified using ImageJ® software, areas of spheroids were quantified, and symbol *($p < 0.05$) indicates a significant difference compared to the third day (D3). Scale bars: 100 µm. Assays were performed in triplicate on three independent experiments.

Table S1: Chemical composition of the GTE

**Compound**	**Content (mg/g)**
Catechin (C)	4.7 ± 0.08
Epicatechin gallate (ECG)	7.8 ± 0.03
Epigallocatechin (EGC)	28.6 ± 0.02
Epigallocatechin-3-gallate (EGCG)	28.4 ± 0.1
∑ catechins	69.5 ± 0.08
